# Therapeutic drug monitoring of atomoxetine in children and adolescents with attention-deficit/ hyperactivity disorder: a naturalistic study

**DOI:** 10.1007/s00702-022-02483-8

**Published:** 2022-04-07

**Authors:** Katrin Ruppert, Christoph Geffert, Hans-Willi Clement, Christian Bachmann, Michael Haberhausen, Eberhard Schulz, Christian Fleischhaker, Monica Biscaldi-Schäfer

**Affiliations:** 1 Department of Child and Adolescent Psychiatry, Psychotherapy and Psychosomatics, Faculty of Medicine, University of Freiburg, Freiburg im Breisgau, Germany; 2 Dr. Staber & Kollegen, Klipphausen, Germany; 3 Department of Child and Adolescent Psychiatry, University Hospital Marburg, Philipps-University Marburg, Marburg, Germany; 4 Department of Child and Adolescent Psychiatry and Psychotherapy, University of Ulm, Ulm, Germany

**Keywords:** Atomoxetine, ADHD, TDM, Children, Serum concentration

## Abstract

The selective norepinephrine reuptake inhibitor atomoxetine is potentially among the first-line pharmacotherapy options for ADHD. Therapeutic drug monitoring (TDM) with the quantification and interpretation of atomoxetine serum concentrations is used to determine an individual dose followed by an optimal effectiveness and minimal side effects. The aim of this retrospective pharmacokinetic–pharmacodynamic analysis was to derive age-appropriate recommendations for the implementation of TDM to improve the efficacy and tolerability of atomoxetine in children and adolescents. Using the analytical method of high-performance liquid chromatography with UV detection, 94 serum concentrations of 74 patients between 6 and 21 years of age were determined. Therapeutic effectiveness and side effects were evaluated according to the categories “low”, “moderate”, and “significant”. As part of TDM, a time interval with maximum concentrations of 1–3 h after the administration of atomoxetine was determined for blood sampling. In this time interval, a significant correlation between the weight-normalized dose and the serum concentrations was found. The efficacy as well as the tolerability proved to be mainly moderate or significant. A preliminary therapeutic reference range was between 100 and 400 ng/ml. Naturalistic studies have limitations. Therefore, and due to a limited study population, the results have to be regarded as preliminary observations that must be confirmed in further studies. The preliminary therapeutic reference range for children and adolescents proved to be narrower than the reference range for adult patients. However, due to good efficacy and tolerability an exact reference range remained difficult to determine.

## Introduction

Atomoxetine is a selective norepinephrine reuptake inhibitor (NARI) and is potentially among the first-line pharmacotherapy options in the treatment of attention-deficit hyperactivity disorder (ADHD), one of the most common psychiatric disorders in children and adolescents (Wong et al. 1982; Sauer et al. 2005; Briars and Todd 2016; Arbeitsgemeinschaft der Wissenschaftlichen Medizinischen Fachgesellschaften 2017; Falkai et al. 2018). Compared to placebo, atomoxetine shows a significantly stronger effect in terms of symptom reduction and improvement of functional abilities in children and adolescents with ADHD (Michelson et al. 2001, 2002; Gayleard and Mychailyszyn 2017). If a therapy with psychostimulants is not sufficiently effective, if side effects occur, contraindications exist or if coexisting disorders are present, a guideline-based therapy considers atomoxetine to be a second-best choice following methylphenidate (Lilly Deutschland Gmbh 2015; Arbeitsgemeinschaft der Wissenschaftlichen Medizinischen Fachgesellschaften 2017; National Institute for Health and Care Excellence 2018). Almost one third of the children who initially received methylphenidate and later on atomoxetine, responded selectively better to one of the two drugs (Newcorn et al. 2008).

However, there are interindividual differences in the pharmacokinetics of atomoxetine; fluctuations in serum concentrations and a different therapeutic response may be the consequence (Hiemke et al. 2012, 2018). Not only parameters such as age, comorbidities, comedications, compliance and smoking, but also genetic polymorphisms can have an influence on pharmacokinetics (Paulzen et al. 2008; Hiemke et al. 2012). With regard to genetic polymorphisms and their influence on the metabolism of atomoxetine, the main focus is on cytochrome P450 enzymes (CYP enzymes), whose activity can be altered by such polymorphisms. The metabolism of atomoxetine to the main metabolite 4-hydroxyatomoxetine is primarily mediated by CYP2D6 (Ring et al. 2002). The side metabolite N-desmethylatomoxetine is mainly catalyzed by CYP2C19 (Ring et al. 2002). The influence of CYP2C19 on the metabolism of atomoxetine is estimated to be small (Sauer et al. 2005; Yu et al. 2016).

Regarding the activity of CYP2D6, "extensive metabolizers" (EM) and "poor metabolizers" (PM) can be distinguished. More than 90% of all individuals, the so-called EM, show a normal activity of CYP2D6 (Sauer et al. 2005; Lilly Deutschland Gmbh 2015). The two CYP polymorphisms lead to clinically relevant differences in the pharmacokinetics of atomoxetine. PM can have up to ten times higher mean serum concentrations than EM; the half-life can increase four to five times (Sauer et al. 2003, 2005; Witcher et al. 2003). Pharmacokinetics can also be influenced by drug interactions. Besides atomoxetine, approximately 25% of all current drugs are metabolized by CYP2D6 and can, therefore, interact with atomoxetine when taken simultaneously (Kirchheiner et al. 2004; Ingelman-Sundberg 2004; Zhou 2009).

To assess the influence of interindividual pharmacokinetic differences on the therapeutic effect and the tolerability of atomoxetine, therapeutic drug monitoring (TDM) may be useful (Walitza et al. 2016). TDM represents a cost-effective, personalized method to optimize psychopharmacotherapy (Jang et al. 2016; Hiemke et al. 2018). The aim is to assess an individual, effective drug dose with the best possible outcome and a minimal risk of side effects (Hiemke et al. 2012; Jang et al. 2016). This can be achieved by quantifying and interpreting serum drug concentrations with regards to individual patients’ characteristics as well as pharmacokinetic aspects of a pharmacon (Hiemke et al. 2018). Serum concentrations which are accompanied by maximum efficacy and minimum side effects lie within the therapeutic reference range (Hiemke et al. 2012, 2018). The therapeutic reference range for atomoxetine, which has so far only been defined for adults, is between 200 and 1000 ng/ml for serum concentrations measured 60–90 min after dosing of 1.2 mg/(kg x day) atomoxetine (Hiemke et al. 2018). For children and adolescents, a therapeutic reference range has not been established yet. Sugimoto et al. showed a lower threshold of 64.6 ng/ml for pediatric patients (Sugimoto et al. 2021). Due to the short half-life of atomoxetine, blood samples for the determination of serum concentrations should be taken at the time of maximum serum concentration *C*_max_ (Hiemke et al. 2018).

Especially in children and adolescents, pharmacokinetic aspects can vary and deviate from those of an adult (Gerlach et al. 2006; Mehler-Wex et al. 2009; Pichini et al. 2009). However, studies on pharmacokinetic characteristics of atomoxetine and other psychotropic drugs in children and adolescents are scarce. Approximately one third of psychotropic drugs in children and adolescents are used off-label (Koelch et al. 2009). In an American study, children with ADHD aged 3–5 years have already shown a high use of off-label drug prescriptions, including atomoxetine (Panther et al. 2017). However, in children younger than 6 years, only non-pharmaceutical interventions are recommended (Taylor et al. 2004; Subcommittee on Attention-Deficit/Hyperactivity Disorder 2011; Arbeitsgemeinschaft der Wissenschaftlichen Medizinischen Fachgesellschaften 2017; National Institute for Health and Care Excellence 2018). A non-evidence-based prescription carries the risk of increased, unpredictable side effects or an ineffective therapy due to altered pharmacokinetics in pediatric patients (Egberts et al. 2014). For these reasons, TDM is always indicated in children and adolescents taking psychotropic drugs (Egberts et al. 2011; Gerlach et al. 2016; Hiemke et al. 2018).

The aim of this retrospective pharmacokinetic–pharmacodynamic analysis was to derive age-appropriate recommendations for the implementation of TDM to improve the efficacy and tolerability of atomoxetine in children and adolescents. The focus is especially on the therapeutic efficacy and side effects to determine a possible therapeutic reference range.

## Subjects and methods

### Study design

Within this retrospective, naturalistic study, 94 atomoxetine serum concentrations were analyzed in the neuropharmacological research laboratory of the Department of Child and Adolescent Psychiatry, Psychotherapy and Psychosomatics in Freiburg. To determine these serum concentrations, venous blood samples were collected from 74 children and adolescents between June 2005 and July 2014. The children and adolescents were treated in the outpatient or inpatient Department of Child and Adolescent Psychiatry, Psychotherapy and Psychosomatics in Freiburg. In addition, some blood samples analyzed in the neuropharmacological research laboratory in Freiburg as part of clinical routine TDM request were also collected from pediatric patients treated in outpatient and inpatient settings of external university departments for child and adolescent psychiatry. All children and adolescents included in this study were diagnosed with ADHD. Since serum concentrations were determined as part of the clinical routine, no written informed consent was required for collecting and analyzing blood samples as part of TDM.

### Data collection

The collection of patient data for the interpretation of the atomoxetine serum concentrations was based on information on request forms. In addition to the patient’s name and date of birth, the request forms contained information on the dose and dose-distribution, the body weight, the last administration of atomoxetine, the last readjustment of dose and the time and date of blood sample. The main diagnosis and further comorbidities after ICD-10, concomitant medications, the use of oral contraception and smoking habits were further required information. The clinical response and the severity of side effects were described by the treating physician using the categories “low”, “moderate”, and “strong/severe”; a standardized questionnaire for a more precise assessment did not exist. The request forms were sent to the laboratory together with the blood samples. However, some blood samples were handed in without the recommended request form. Additionally, there was a lack of information on some request forms. For this reason, the number of serum concentrations with complete information on the request forms was limited.

### Sample preparation

After the collection of the venous blood sample, the standardized preparation was performed. To obtain serum, blood was centrifuged at 4000 rpm for 10 min. The supernatant was transferred into a new vial. Afterwards, the samples could either be analyzed immediately or be kept at − 80 °C until analysis. The serum of the external samples was obtained on site and then transported to the neuropharmacological research laboratory in Freiburg for further analysis. Serum concentrations were analyzed by high-performance liquid chromatography with ultraviolet detection (UV-HPLC).

### Quantification of atomoxetine

For the quantification of atomoxetine, the serum first had to be purified. For this purpose, 100 μl of the serum was enriched with 10 μl of the internal standard D-clomipramine (500 ng/ml), 10 μl water and 100 μl methanol. After pre-texturing, the solution was centrifuged at 9000 U for 15 min and the supernatant was placed in a new vial. An autoinjector was used to inject a volume of 50 μl of the sample into the chromatographic system and to start the operation cycle. The control sample was analyzed in the same way as the patient sample and aimed to detect measurement variations. It contained 100 μl serum of a healthy patient without the intake of atomoxetine, 10 μl atomoxetine in a defined concentration, 10 μl of the internal standard D-clomipramine and 100 μl methanol. The internal standard D-clomipramine was added to each sample in a defined concentration as a relative reference size (Funk et al. 2005). The chromatographic system was comprised of an Agilent 1100 series RP-HPLC apparatus with UV detection (BioRad, Waldbronn, Germany). In total, the operation cycle took 25 min. After the start of the operation cycle with the auto-injection, the sample passed the extraction column (LiChrospher CN 20 µm, MZ-Analysentechnik, Mainz, Germany) at a flow rate of 1.5 ml/min for 7 min using a washing eluent containing 90% water and 10% acetonitrile. The aim was the purification and removal of interfering components. In the subsequent phase, which lasted 10 min, the sample was directed to a second pump. At a flow rate of 1.0 ml/min, the sample passed the analytical column (LiChroCART 125–4 HPLC cartridge) with a stationary phase (LiChrospher 60 RP-select 5 µm, Merck, Darmstadt, Germany) and was separated substrate specific. The eluent in the analytical column consisted of 640 ml 40 mM Na2HPO4 (pH 3.35) and 310 ml acetonitrile. After 17 min both pumps were re-equilibrated. UV light with a wavelength of 214 nm was used for the detection of atomoxetine. The retention time of atomoxetine was 12.3 min, that of D-clomipramine 16.6 min.

For the UV-HPLC method, linearity (*r* > 0.9999) was confirmed in a range from 5 to 2000 ng/ml. The lower limit of determination was 5 ng/ml. The intraday variance was between 2 and 4.3% depending on the concentration of atomoxetine.

### Data analysis

For statistical analysis, calculations were performed with Microsoft Excel^®^ (Version 16, Microsoft, Microsoft Deutschland GmbH, Munich) and SigmaPlot^®^ (Version 14, Systat Software GmbH, Erkrath). Graphs were created with Sigmaplot^®^.

Values were presented as mean value MW ± standard deviation SD. The range was defined by the minimum and maximum value (min–max). For parametrically distributed data, correlations between two variables were calculated using the Pearson correlation coefficient *r*_P_. For non-parametric distributions, the Spearman rank correlation coefficient *r*_S_ was calculated. Low correlations were defined as *r* < 0.5, moderate correlations as *r* = 0.5 to *r* = 0.8 and strong correlations as *r* > 0.8. The presence of a normal distribution was tested by Shapiro–Wilk. Possible differences of two unrelated samples coming from a normally distributed collective were tested by the two-sided *t* test. The significance level was set at *α* = 0.05. For more than two independent samples, the Kruskal–Wallis test was used for analysis of variance. To compare serum concentrations present at a specific time after atomoxetine intake, serum concentrations were backcalculated using the following formula to the time point of *x* (in hours) after atomoxetine intake: concentration at *x* = concentration at time of blood collection/e^(− 0.693 × (Δ*t* between last dose and blood collection − *x*)/*t*_1/2_) with *t*_1/2_ = 3.6 h for a EM according to the product characteristics of Strattera® (Bauer 2014; Lilly Deutschland Gmbh 2015; Hiemke et al. 2018). Serum concentrations of 0 ng/ml could not be backcalculated and had to be excluded.

## Results

### Study population

94 atomoxetine serum concentrations of 74 children and adolescents (88 boys, 6 girls) aged between 6 and 21 years were available for the analysis of this study (Table 1). Due to incomplete information on the request forms, the following analysis could only be described for a limited population. The average body weight was 45.4 ± 18.8 kg (*n* = 78), the average daily dose was 42.9 ± 18.6 mg/day (*n* = 82). An ICD-10 diagnosis was given for 44 of the 74 children and adolescents. 16 children and adolescents additionally suffered from up to three comorbid disorders. A comedication was taken by 22 children. None of the pediatric patients used oral contraception. Three adolescents reported nicotine abuse.

**Table 1:** Characteristics of study population (*n* = 94)

Characteristics of study population (*n* = 94)		
Serum concentrations, *n*	94	
Patients, *n*	74	
Gender, *n* (%)		
Male	88	(93.6)
Female	6	(6.4)
Age (years), *n* = 93, mean ± SD (min–max)	11.6 ± 3.3	(6–21)
Weight (kg), *n* = 78, mean ± SD (min–max)	45.4 ± 18.8	(21.6–91.2)
Nicotine abuse, *n* (%)	3	(3,2)
Oral contraception, *n* (%)	0	(0)
Dose (mg/day), *n* = 82, mean ± SD (min–max)	42.9 ± 18.6	(10–80)
Weight-normalized dose (mg/(kg x day)), *n* = 76, mean ± SD (min–max)	1.0 ± 0.3	(0.3–2.1)
Patients with ICD-10 Diagnosis, *n* (%)	44	
Attention deficit hyperactivity disorder, predominantly inattentive type (F90.0)	18	(41)
Attention deficit hyperactivity disorder, predominantly hyperactive type (F90.1)	22	(50)
Combined vocal and multiple motor tic disorder (F95.2)	3	(7)
Overactive disorder associated with mental retardation and stereotyped movements	1	(2)
Patients with comorbidities, *n* (%)	16	
One comorbidity	11	(68.8)
More than one comorbidity	5	(31.2)
Comorbidities, *n*, multiple entries		
Undifferentiated schizophrenia (F20.3)	1	
Major depressive disorder, moderate (F32.1)	1	
Adjustment disorders (F43.2)	1	
Anorexia nervosa (F50.0)	1	
Sleep terrors (F51.4)	1	
Other developmental disorders of speech and language (F80.8)	1	
Specific reading disorder (F81.0)	3	
Mixed disorder of scholastic skills (F81.3)	1	
Specific developmental disorder of motor function (F82.9)	1	
Atypical autistic disorder (F84.1)	1	
Asperger’s syndrome (F84.5)	1	
Other mixed disorders of conduct and emotions (F92.8)	3	
Other childhood emotional disorders (F93.8)	2	
Childhood emotional disorder, unspecified (F93.9)	1	
Enuresis not due to a substance or known physiological condition (F98.0)	1	
Encopresis not due to a substance or known physiological condition (F98.1)	1	
Other abnormal auditory perceptions (H93.2)	1	
Patients with comedication, *n* (%)	22	
One comedication	19	(86.4)
More than one comedication	3	(13.6)
Comedication, *n* (%), multiple entries		
Clozapine	1	(4)
Lamotrigine	1	(4)
Lisdexamphetamine	1	(4)
Methylphenidate	9	(36)
Olanzapine	1	(4)
Pipamperone	2	(8)
Propiverine	1	(4)
Risperidone	7	(28)
Sodium valproate	1	(4)
Sulpiride	1	(4)
Therapeutic efficacy, *n* (%)	60	
Low efficacy	5	(8.3)
Moderate efficacy	28	(46.7)
Strong efficacy	27	(45)
Side effects, *n* (%)	56	
Severe side effects	8	(14.3)
Moderate side effects	5	(8.9)
Minor/no side effects	43	(76.8)

*SD* standard deviation, *min* minimum, *max* maximum, *ICD-10* International statistical classification of diseases and related health problems

For a population of *n* = 27 all parameters necessary to evaluate the pharmacokinetics of atomoxetine were available. As Witcher et al. (2003) showed in their pharmacokinetic analyses for children and adolescents, parameters including age, body weight, dose and especially the time interval between atomoxetine intake and blood collection were indispensable for this purpose. Only one serum concentration per patient was included for the population of *n* = 27, therefore, the data did not need to be corrected for multiple inclusion. If multiple measurements were obtained from one patient, the concentration that was within the time window of 1–4 h with maximum serum concentrations according to Witcher et al. (2003) was selected. The characteristics of the population of *n* = 27 are outlined in Table 2. In this population, children ≤ 12 years of age took a significantly lower absolute dose (37.4 ± 16.0 mg/day) than children > 12 years of age (60.0 ± 16.1 mg/day) (*p* = 0.001). The weight-normalized dose in the age group ≤ 12 years was, however, higher than in the age group > 12 years (*p* = 0.076). A diagnosis was documented for 20 of the 27 children and adolescents. The most frequent diagnoses according to ICD-10 were predominantly inattentive attention-deficit hyperactivity disorder (ADHD) (F90.0) in combination with other comorbid diagnoses (*n* = 7), followed by predominantly hyperactive attention-deficit hyperactivity disorder (ADHD) (F90.1) (*n* = 6). 12 patients suffered from further comorbid disorders. Seven children and adolescents reported a comedication. Among the concomitant medications, methylphenidate and risperidone were the ones administered most frequently.

**Table 2:** Characteristics of limited study population (*n* = 27)

Characteristics of limited study population (*n* = 27)		
Serum concentrations, *n*	27	
Patients, *n*	27	
Gender, *n* (%)		
Male	26	(96.3)
Female	1	(3.7)
Age (years), *n* = 27, mean ± SD (min–max)	12 ± 3.4	(8–21)
Children ≤ 12 years, *n* (%)	14	(51.9)
Adolescents > 12 years, *n* (%)	13	(48.1)
Weight (kg), *n* = 27, mean ± SD (min–max)	49.5 ± 20.2	(21.9–85)
Nicotine abuse, *n* (%)	3	(11.1)
Oral contraception, *n* (%)	0	(0)
Dose (mg/day), *n* = 27, mean ± SD (min–max)	48.2 ± 19.4	(10–80)
Weight-normalized dose (mg/(kg x day)), *n* = 27, mean ± SD (min–max)	1.0 ± 0.3	(0.3–1.6)
Patients with ICD-10 diagnosis, *n* (%)	20	
Attention deficit hyperactivity disorder, predominantly inattentive type (F90.0)	8	(40)
Attention deficit hyperactivity disorder, predominantly hyperactive type (F90.1)	10	(50)
Combined vocal and multiple motor tic disorder (F95.2)	2	(10)
Patients with comorbidities, *n* (%)	12	
One comorbidity	10	(83.3)
More than one comorbidity	2	(16.7)
Comorbidities, *n*, multiple entries		
Undifferentiated schizophrenia (F20.3)	1	
Major depressive disorder, moderate (F32.1)	1	
Anorexia nervosa (F50.0)	1	
Sleep terrors (F51.4)	1	
Specific reading disorder (F81.0)	3	
Specific developmental disorder of motor function (F82.9)	1	
Atypical autistic disorder (F84.1)	1	
Other mixed disorders of conduct and emotions (F92.8)	2	
Other childhood emotional disorders (F93.8)	2	
Childhood emotional disorder, unspecified (F93.9)	1	
Patients with comedication, *n* (%)	7	
One comedication	6	(85.7)
More than one comedication	1	(14.3)
Comedication, *n* (%), multiple entries		
Clozapine	1	(12.5)
Methylphenidate	2	(25)
Olanzapine	1	(12.5)
Pipamperone	1	(12.5)
Risperidone	2	(25)
Sodium valproate	1	(12.5)

*SD* standard deviation, *min* minimum, *max* maximum, *ICD-10* International statistical classification of diseases and related health problems

### Relationship between serum concentration and time of collection

The mean value of all 94 serum concentrations was 203.4 ± 293.0 ng/ml (0–1625 ng/ml). The 27 serum concentrations with complete information on pharmacokinetic parameters ranged from 0 to 1334 ng/ml (mean value 213.9 ± 277.8 ng/ml). The spectrum of the 27 serum concentrations normalized to 1 mg/kg ranged from 0 (ng/ml)/(mg/kg) to 1037.6 (ng/ml)/(mg/kg) (mean value 207.0 ± 244.1 (ng/ml)/(mg/kg)).

Time intervals between the intake of atomoxetine and the time of blood collection were distributed from 1 to 36 h for *n* = 27 (mean value 10.4 ± 9.8 h). The relationship between serum concentrations and time intervals showed a rapid absorption with peak concentrations in the first few hours after intake. As time proceeded, serum concentrations decreased. 37% of the serum concentrations were within the therapeutic reference range of 200–1000 ng/ml valid for adult patients (Hiemke et al. 2018). 59% of the serum concentrations were below the therapeutic reference range—most of them were associated with advanced time intervals.

Witcher et al. (2003) presented pharmacokinetic analyses of atomoxetine for 16 children and adolescents with the genotype of an EM and determined a time window of maximum serum concentrations between 1 and 4 h after the intake. In this study, ten measurements were taken within the time interval of 1–4 h. Low serum concentrations beyond the 95% confidence interval were excluded due to a potential non-compliance (*n* = 2). Weight-normalized concentrations ranged from 146.9 to 467.9 (ng/ml)/(mg/kg) in this time interval (*n* = 10). A comparison of weight-normalized serum concentrations in the time interval of 1–4 h (299.1 ± 116.3 (ng/ml)/(mg/kg), *n* = 10) with the subsequent time interval of 5–9 h (155.6 ± 46.3 (ng/ml)/(mg/kg), *n* = 4) showed a significant difference (*p* = 0.006) using *t* test. A normal distribution according to Shapiro–Wilk was present.

For a better comparison of serum concentrations within the time window of 1–4 h, concentrations with a given time interval were backcalculated to time points of 1 h, 1.5 h, 3 h and 4 h after atomoxetine intake. Serum concentrations of 0 ng/ml could not be backcalculated. Three outliers with concentrations beyond 1.5 times the interquartile range on the boxplot were excluded as potentially resulting from PM. Their concentrations at the time point of 1.5 h were 14,797.1 ng/ml, 243,604.5 ng/ml and 1619.5 ng/ml. The mean values of the remaining serum concentrations were 381.3 ± 238.3 ng/ml (*n* = 20) at 1 h, 346.3 ± 216.4 ng/ml (*n* = 20) at 1.5 h, 259.5 ± 162.2 ng/ml (*n* = 20) at 3 h and 214.0 ± 133.8 ng/ml (*n* = 20) at 4 h after atomoxetine intake. A peak concentration 1 h after intake with a subsequently decreasing concentration curve could be demonstrated. Serum concentrations at the time point of 4 h were already significantly lower than concentrations at the time point of 1 h and 1.5 h (*p* = 0.010 or *p* = 0.027).

### Relationship between serum concentration and daily dose

In the time interval between 1 and 4 h after the intake of atomoxetine, a moderate correlation according to Pearson was shown for the relationship between serum concentrations and the absolute daily dose (*r*_P_ = 0.534, *p* = 0.112, *n* = 10). Between serum concentrations and the relative, weight-normalized daily dose a stronger, significant correlation was observed (*r*_P_ = 0.632, *p* = 0.050, *n* = 10). The parameters were normally distributed. After the additional exclusion of patients with comedication or nicotine abuse to rule out an interaction with these potentially interfering variables, a significant correlation of *r*_P_ = 0.807 was found (*r*_P_ = 0.807, *p* = 0.028, *n* = 7).

After backcalculating all serum concentrations to the time point of 90 min after atomoxetine intake, a significant, moderate correlation between serum concentrations at the time point of 90 min and the weight-normalized daily dose could be confirmed (*r*_P_ = 0.482, *p* = 0.030, *n* = 20). After excluding patients with comedication or nicotine abuse and low serum concentrations with possible non-compliance a significant, moderate correlation was supported (*r*_P_ = 0.718, *p* = 0.013, *n* = 11).

### Relationship between serum concentration and therapeutic efficacy

Data on the efficacy of atomoxetine were available for 60 serum concentrations. On the request form, categories of a “low”, “moderate” and “strong” efficacy were distinguished. 27 children noted a marked, 28 a moderate improvement of their symptoms. Only five reported little or no effect.

**Fig. 1:**
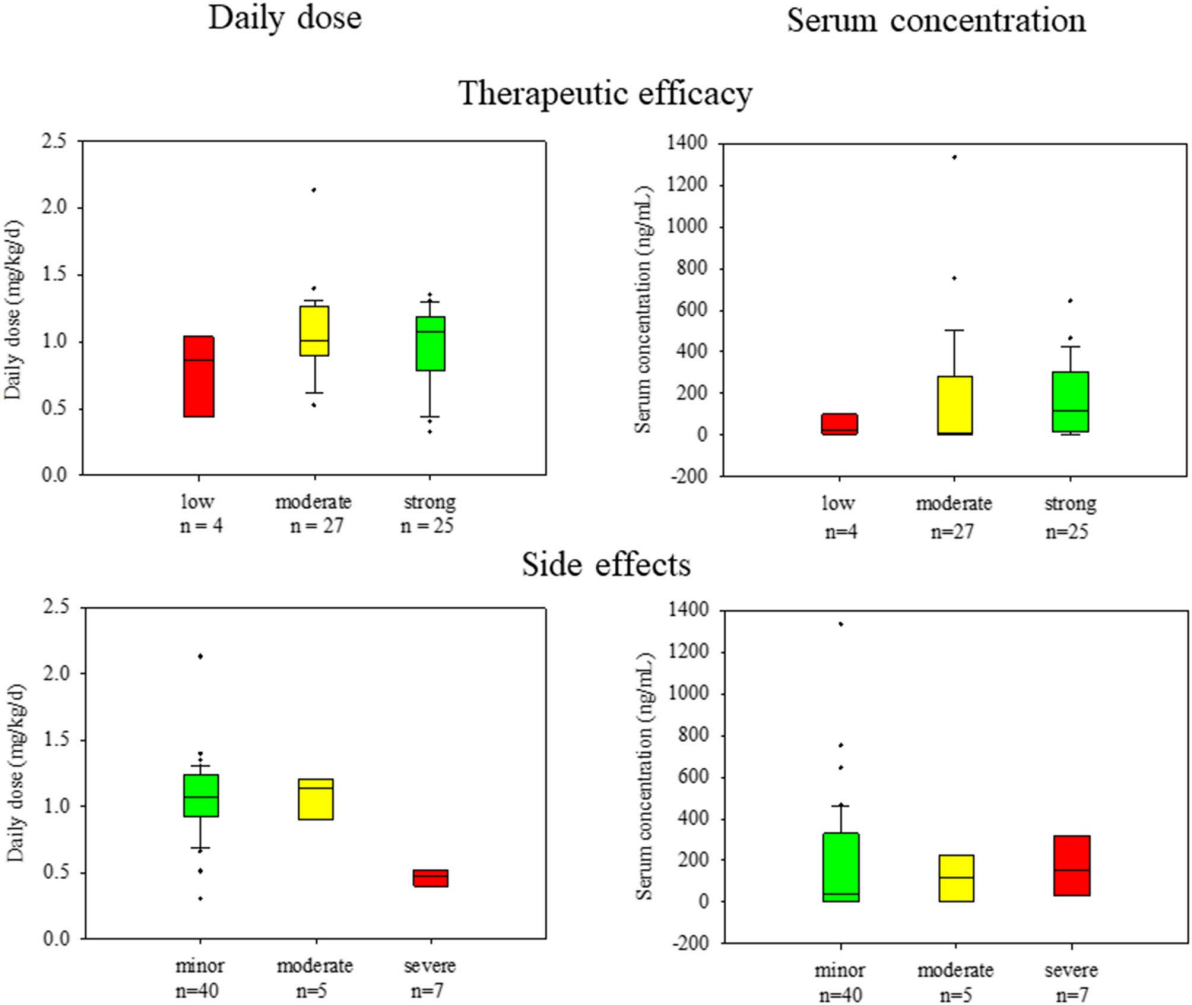
Boxplot with weight-normalized doses as well as serum concentrations related to therapeutic efficacy, *n* = 56, and side effects, *n* = 52. Boxplot: lower limit of the box = 25th percentile; upper limit of the box = 75th percentile; line inside the box = median; whisker below the box = 10th percentile; whisker above the box = 90th percentile; dots = outlier

With regard to serum concentrations and the weight-normalized daily dose, potential differences between these three categories were investigated. The mean value for serum concentrations with little effect on symptom reduction was 43 ± 56.4 ng/ml (*n* = 4), for concentrations with a moderate effect 176.5 ± 299.8 ng/ml (*n* = 27) and for concentrations with a strong effect 173.5 ± 176.9 ng/ml (*n* = 25). The corresponding median values and interquartile ranges are listed in Table 3. Using Kruskal–Wallis test, no significant difference was found between the three categories (*X*^2^ = 2.4; *df* = 2; *p* = 0.299). In contrast, the comparison of just two categories using *t* test showed significant differences not only between the serum concentrations with a low and moderate effect (*p* = 0.047) but also between the concentrations with a low and strong effect (*p* = 0.011) (Table 3). Comparatively lower serum concentrations thus seemed to be associated with a low efficacy, higher serum concentrations with a moderate and strong efficacy (Fig. 1).

**Table 3:**
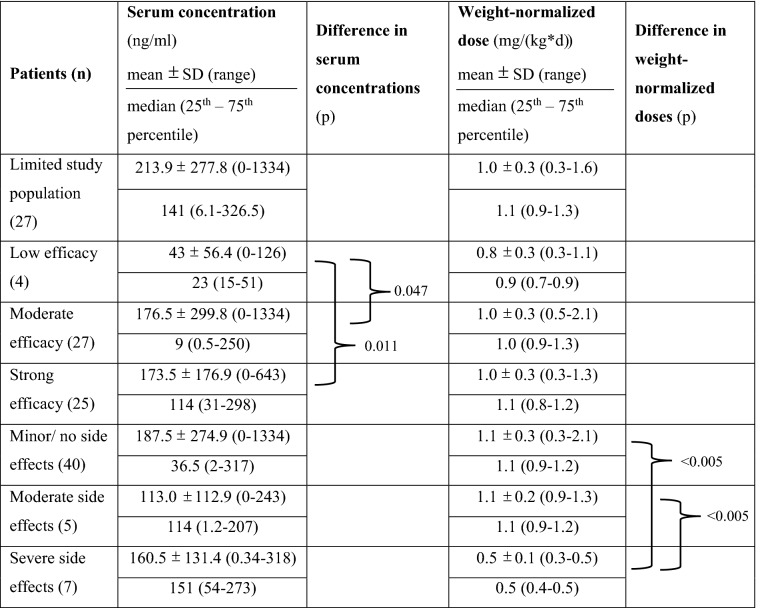
Serum concentrations, weight-normalized daily doses and their differences in relation to therapeutic efficacy and side effects of atomoxetine

Differences were calculated using *t *test

Regarding the relationship between weight-normalized daily dose and efficacy, no significant difference could be observed (*X*^2^ = 2.2; *df* = 2; *p* = 0.332). The mean value of the weight-normalized daily dose with a moderate and strong effect on symptom reduction was 1.0 ± 0.3 mg/(kg x day), with a low efficacy 0.8 ± 0.3 mg/(kg x day) (Table 3, Fig. 1).

To consider the time interval between the intake and the time of blood collection, 19 serum concentrations with a known efficacy could be backcalculated to the time point of 90 min after atomoxetine intake. A concentration with 440.3 ng/ml at the time point of 90 min showed low efficacy (*n* = 1). The mean value for concentrations with moderate efficacy was 1818.2 ± 4566.8 ng/ml (*n* = 10) and with strong efficacy 248.4 ± 153.4 ng/ml (*n* = 8). The high mean value and standard deviation for concentrations with moderate efficacy could be explained by an outlier with a concentration of 14797.1 ng/ml. Excluding the outlier, the mean value was 376.1 ± 257.2 ng/ml (*n* = 9). The median value with the interquartile range from the 25th to the 75th percentile was 456.7 ng/ml with a range from 202.2 to 653.3 ng/ml for concentrations with moderate efficacy and 257.2 ng/ml with a range from 192.0 to 306.8 ng/ml for concentrations with strong efficacy. Considering the time interval, no difference between the concentrations with a low, moderate or strong effect could be demonstrated (*X*^2^ = 2.3; *df* = 2; *p* = 0.321). Therefore, a correlation between the level of serum concentration and efficacy could not be verified, when taking the time interval into account.

After excluding the outlier, all mean values as well as all median values with the interquartile ranges were within the therapeutic reference range originally being valid for adult patients (Hiemke et al. 2018). Regarding the individual concentrations, few concentrations going along with a moderate or strong efficacy were below the therapeutic range given by Hiemke et al. Only the outlier with a moderate efficacy was above the range.

The mean values for weight-normalized doses at the time point of 90 min with a low, moderate and strong effect were 1.1 mg/(kg x day) (*n* = 1), 1.1 ± 0.3 mg/(kg x day) (*n* = 10) and 1.0 ± 0,3 mg/(kg x day) (*n* = 8); a difference could not be shown using Kruskal–Wallis (*X*^2^ = 2.56; *df* = 2; *p* = 0.880).

A therapeutic reference range with a defined upper and lower threshold for children and adolescents does not yet exist. According to the consensus guidelines, the arithmetic mean ± standard deviation of serum concentrations at which a psychotropic drug is well effective can serve as a preliminary reference range in the absence of a therapeutic reference range (Hiemke et al. 2018). Therefore, serum concentrations with a strong effect at the time point of 90 min after atomoxetine intake were evaluated (*n* = 8) and a therapeutic reference range of 248 ng/ml ± 153 ng/ml, i.e. approximately from 100 to 400 ng/ml, could be suggested. As concentrations with known efficacy were not normally distributed, the interquartile range of serum concentrations with a strong effect at the time point of 90 min was additionally calculated to provide another possible therapeutic reference range (Bengtsson 2004). The 25th and the 75th percentile of the interquartile range were 192.0 and 306.8 ng/ml (*n* = 8). Hence, using the interquartile range, the therapeutic reference range from approximately 200 to 300 ng/ml was narrower.

### Relationship between serum concentration and side effects

Side effects were reported for 56 serum concentrations. Whereas severe side effects were described for eight measurements, five measurements were accompanied by moderate side effects. In most cases (*n* = 43), there were no or just minor side effects.

Irrespective of the degree of side effects, all serum concentrations were in a similar range. Using Kruskal–Wallis, no significant difference was found between the serum concentrations with minor, moderate or either severe side effects (*X*^2^ = 0.59; *df* = 2; *p* = 0.743). Children with minor side effects had an average serum concentration of 187.5 ± 274.9 ng/ml (*n* = 40). The mean values of serum concentrations with moderate and severe side effects were 113.0 ± 112.9 ng/ml (*n* = 5) and 160.5 ± 131.4 ng/ml (*n* = 7) (Fig. 1). The corresponding median values and interquartile ranges are listed in Table 3.

Analysis of weight-normalized doses showed a significant difference depending on the degree of side effects (*X*^2^ = 15.78; *df* = 2; *p* = 0.0004). The mean value of weight-normalized doses accompanied by minor side effects was 1.1 ± 0.3 mg/(kg x day) (*n* = 40). For those with moderate side effects the mean value was 1.1 ± 0.2 mg/(kg x day) (*n* = 5) and for those with severe side effects 0.5 ± 0.1 mg/(kg x day) (*n* = 7) (Table 3). Thus, despite significantly lower weight-normalized doses for children with severe side effects, their serum concentrations were comparable with those for patients with minor or moderate side effects. For children and adolescents with moderate and minor side effects, serum concentrations seemed to be associated with higher doses (Fig. 1).

To consider the time interval for the analysis of side effects, 20 serum concentrations could be backcalculated to the time point of 90 min after atomoxetine intake. The mean value for serum concentrations with minor side effects was 1329.9 ± 3731.3 ng/ml (*n* = 15) including an outlier with a serum concentration of 14,797.1 ng/ml. The corresponding median value was 368.8 ng/ml with an interquartile range from 239.2 to 565.9 ng/ml (*n* = 15). For concentrations with moderate side effects, the mean value was 282.8 ± 16.6 ng/ml (*n* = 2) and the median value was 282.8 ng/ml with an interquartile range from 277.0 to 288.7 ng/ml (*n* = 2). For concentrations with severe side effects, the mean value was 81233.4 ± 140617.5 ng/ml (*n* = 3) including an outlier with a serum concentration of 243694.5 ng/ml. The median value was 69.9 ng/ml with an interquartile range from 47.9 to 121837.2 ng/ml (*n* = 3). A significant difference between these three groups could still not be demonstrated (*X*^2^ = 0.45; *df* = 2; *p* = 0.798).

The mean values of concentrations with minor as well as with severe side effects were above the therapeutic range between 200 and 1000 ng/ml valid for adult patients (Hiemke et al. 2018). The high mean values resulted from two extreme high concentrations, of which only the higher one (243694.5 ng/ml) was associated with severe side effects. A comedication or nicotine abuse did not exist. Excluding both outliers, the mean values were 367.9 ± 213.7 ng/ml for concentrations with minor side effects (*n* = 14) and 47.9 ± 31.2 ng/ml for concentrations with severe side effects (*n* = 2). The other two concentrations with severe side effects were thus below the therapeutic reference range.

As already suspected, analysis of weight-normalized doses at the time point of 90 min confirmed a significant difference depending on the degree of side effects (*X*^2^ = 8.09; *df* = 2; *p* = 0.018). The mean value of weight-normalized doses going along with minor side effects was 1.1 ± 0.2 mg/(kg x day) (*n* = 15) and with moderate side effects 1.0 ± 0.2 mg/(kg x day) (*n* = 2). Doses with severe side effects had a mean value of only 0.5 ± 0.03 mg/(kg x day) (*n* = 3) (Table 3).

## Discussion

This retrospective pharmacokinetic–pharmacodynamic analysis is one of the few studies examining the pharmacokinetics of atomoxetine in children and adolescents. Although the level of recommendation for TDM in children and adolescents corresponds to a level one with a strong recommendation of an implementation, only few studies exist for this specific age group (Hiemke et al. 2018). In the consensus guidelines, recommendations for therapeutic reference ranges have so far only applied to adult patients (Hiemke et al. 2018). The result is a discrepancy between evidence and prescription frequency in children and adolescents. Since blood should be withdrawn at the time of maximum drug concentration *C*_max_ in the context of TDM, one focus of this study was the evaluation of a time interval for blood collection for children and adolescents (Hiemke et al. 2018). Furthermore, the relationship between dose and serum concentrations and, in particular, efficacy and tolerability were evaluated to verify an age-appropriate therapeutic reference range.

In one of the few available studies for children and adolescents, Witcher et al. (2003) examined the pharmacokinetic characteristics in a collective of 21 children and adolescents. According to Witcher et al. (2003), maximum serum concentrations were within a time interval of 1–4 h after the intake of atomoxetine. In this study, serum concentrations normalized to 1 mg/kg could be well classified into the pharmacokinetic profiles of Witcher et al. (2003). An extension of the time interval was not reasonable. In the time interval of 5–9 h, serum concentrations dropped to significantly lower levels. However, when correcting concentrations for defined time points of 1 h, 1.5 h, 3 h and 4 h, significantly lower serum concentrations could already be verified at 4 h compared to 1 h and 1.5 h after atomoxetine intake. In the consensus guidelines valid for adults, a narrower time interval of 60–90 min was described for blood collection (Hiemke et al. 2018). The time interval of 60–90 min was also used in several studies (Farid et al. 1985; Michelson et al. 2007; Hazell et al. 2009). Sauer et al. supported a time interval of 1–3 h (Sauer et al. 2003). In this study, peak concentrations could be confirmed at 60 min and 90 min after atomoxetine intake, concentrations at 3 h were still high but already decreased. Yet, a significant difference between concentrations at 1 h or 1.5 h and 3 h was not present. A narrow time interval of 60–90 min is difficult to implement for children and adolescents in daily life. The risk of non-compliance could be a consequence. An extension of the time window to perform blood collection would be easier to implement due to a greater flexibility in everyday clinical practice. Therefore, a wider interval between 1–3 h could be considered for children and adolescents. Since concentrations were significantly lower 4 h after atomoxetine intake, an extension to a time window of 1–4 h cannot be recommended.

The range of time intervals from 1 to 36 h was wide and correlated with the large variability of serum concentrations from 0 to 1334 ng/ml. More than 50% of the 27 serum concentrations were based on time intervals of more than 4 h. The wide range suggests that a time interval for blood collection has not yet been sufficiently established in clinical routine.

The moderate to strong correlation between serum concentrations and weight-normalized dose—as opposed to the lower correlation between serum concentrations and absolute dose—supports the recommendation of a weight-normalized dosing of atomoxetine for children and adolescents (Lilly Deutschland Gmbh 2015). Hereby, not only comparability between children and adolescents is achieved, but also a constant exposure to atomoxetine during childhood is ensured (Sauer et al. 2005). The observation that children ≥ 12 years of age received a higher absolute but lower weight-normalized dose compared to children and adolescents < 12 years of age shows that the absolute dose is underestimated for older children. Due to an older age, there is a risk of prescribing a higher absolute but not yet sufficiently high weight-normalized dose.

As already mentioned, our analysis showed a significantly moderate to strong correlation between serum concentrations and weight-normalized dose. Comparable correlations have not yet been described for atomoxetine; so far, only a proportionality between the weight-normalized dose and the AUC of atomoxetine for children and adolescents (Witcher et al. 2003) and for adult patients (Farid et al. 1985) has been described in literature. In various studies, correlations between dose and serum concentrations in children and adolescents were examined for other psychotropic drugs (Schulz et al. 1995; Gerlach et al. 2007; Koelch et al. 2012; Taurines et al. 2013; Wohkittel et al. 2016). A comparably strong correlation (*r*_P_ = 0.807, *p* = 0.0001) was observed for clozapine (Schulz et al. 1995).

However, a disadvantage of this analysis was the small number of serum concentrations due to the exclusion of measurements with incomplete information on the request forms and with a possible CYP polymorphism or possible confounding variables such as a comedication or non-compliance.

In the time interval of maximum concentrations, for example, significantly lower serum concentrations outside the 95% confidence interval were excluded due to potential non-compliance. A comedication or nicotine abuse could be excluded for these concentrations. Also, an altered CYP-metabolism was unlikely; the absorption of atomoxetine does not differ between EM and PM, both show maximum serum concentrations about 2 h after the intake of atomoxetine (Sauer et al. 2003). Therefore, non-compliance was most likely to be assumed.

After backcalculating concentrations to the time point of 90 min, three outliers with extreme high serum concentrations above 1.5 times the interquartile range on the boxplot also had to be excluded. Even without backcalculating these three outliers, their concentrations were high according to their advanced time intervals between 13 and 36 h (1334 ng/ml after 14 h, 318 ng/ml after 36 h and 177 ng/ml after 13 h). For these patients, an altered CYP-metabolism as that of PM could be suspected. PM can have up to ten times higher mean atomoxetine concentrations as well as four to five times increased half-lives compared to EM (Farid et al. 1985; Sauer et al. 2003, 2005; Brown et al. 2016; Lilly Deutschland Gmbh 2015). In the case of PM, however, the backcalculation formula, which used a half-life of 3.5 h assuming an EM, is not transferable and the three concentrations at 90 min are likely overestimated. Whether the formula can be applied to all other concentrations with the pharmacokinetics of children and adolescents cannot be answered with certainty.

Besides, measurements with a comedication or a nicotine abuse were excluded. Potential CYP-interactions due to a comedication can have an influence on atomoxetine serum concentrations (Paulzen et al. 2008; Hiemke et al. 2018). Risperidone, for example, is metabolized via CYP2D6, as is atomoxetine. By inhibiting the catalytic activity of CYP2D6, risperidone could lead to a potential increase in the concentration of atomoxetine (Shin et al. 1999; Belle et al. 2002; Ring et al. 2002). A relevant inhibitory effect of antipsychotics such as risperidone, however, was neither observed in this nor in other studies (Ring et al. 1996; Shin et al. 1999). Nicotine may exert a small inductive effect on CYP1A2 and CYP2E1. In small but not relevant parts, atomoxetine could also be metabolized by these CYP enzymes (Zevin and Benowitz 1999; Ring et al. 2002; Miksys and Tyndale 2006; Mann et al. 2008). Because of their potential interactions—albeit small and clinically irrelevant—measurements with a comedication and a nicotine abuse were excluded to guarantee valid results.

Overall, our study showed a good efficacy of atomoxetine. In literature, numerous studies or meta-analyses confirmed an equally effective response to atomoxetine in children and adolescents with ADHD (Kratochvil et al. 2001; Spencer et al. 2001; Michelson et al. 2001; Buitelaar et al. 2004; Bakken et al. 2008; Gayleard and Mychailyszyn 2017). The detailed analyses of these studies were often based on the “CGI Severity Scale CGI-S” or the ADHD Rating Scale (Spencer et al. 2001; Michelson et al. 2001, 2007). In this study, however, the analysis was only based on the categories of a low, moderate or strong efficacy according to the less detailed information on the request form.

Initially, efficacy seemed to be independent of the weight-normalized dose, but dependent on serum concentrations. After backcalculating concentrations to the time point of 90 min, this thesis had to be refuted. Neither a concentration effect relationship nor a dose effect relationship could be recorded. Concentrations with a moderate and strong effect were mainly within but also below the therapeutic range valid for adults (Hiemke et al. 2018). Analyses in literature support this. Low weight-normalized doses of 0.5 mg/(kg x day) were associated with a low efficacy (Michelson et al. 2001; Trzepacz et al. 2008). An increase of the weight-normalized dose beyond the recommended maintenance dose of 1.2 mg/(kg x day), however, showed no further improvement of efficacy (Michelson et al. 2001; Kratochvil et al. 2007; Hazell et al. 2009). Regarding the lack of a concentration effect relationship, Hazell et al. (2009) confirmed that clinical efficacy of atomoxetine could not be predicted by the level of serum concentrations. Also, Michelson et al. confirmed a nonlinear correlation between serum concentrations and efficacy (*r*_P_ = 0.179) (Michelson et al. 2007). Regardless of serum concentrations, a sustained efficacy with a reduction of symptoms over the course of the day was claimed by Kelsey et al. (2004) and Michelson et al. (2002). Correspondingly, a correlation between serum concentrations and their efficacy could not be shown for other psychotropic drugs (Klampfl et al. 2010; Koelch et al. 2012; Taurines et al. 2013; Wohkittel et al. 2016).

Looking more closely at the concentration associated with a low efficacy, the original concentration with a weight-normalized dose of 1.1 mg/kg was 126 ng/ml at the time point of 8 h after atomoxetine intake. After backcalculation to the time point of 90 min, the concentration was 440.3 ng/ml and, therefore, within the therapeutic reference range valid for adults (Hiemke et al. 2018). Thus, the concentration corresponded to the regular pharmacokinetic profiles of atomoxetine. Due to the short half-life of atomoxetine, the concentration could be below the therapeutic reference range 8 h after the intake (Witcher et al. 2003; Sauer et al. 2005). Michelson et al. confirmed that 25% of children and adolescents did not achieve a sufficient efficacy despite a sufficient dose and the presence of an EM (Michelson et al. 2007).

Due to the overall good efficacy regardless of the level of serum concentrations, the definition of a therapeutic reference range for children and adolescents was difficult. Nevertheless, an attempt was made to calculate an age-appropriate therapeutic reference range on the basis of serum concentrations with a strong efficacy in the time interval of maximum serum concentrations. Compared to the therapeutic reference range of 200 to 1000 ng/ml valid for adult patients (Hiemke et al. 2018), the calculated range of 100–400 ng/ml or even of 200–300 ng/ml for children and adolescents was narrower. To achieve a strong effect on symptom reduction in children and adolescents, an upper limit of 1000 ng/ml did not seem necessary. Using ROC analysis, Sugimoto et al. showed a low threshold of 64.6 ng/ml for a possible therapeutic reference range for pediatric patients. When exceeding this plasma concentration of 64.6 ng/ml, the treatment with atomoxetine was more likely to be effective (Sugimoto et al. 2021). This analysis of Sugimoto et al., therefore, supports a lower threshold for children and adolescents than originally assumed for adult patients.

Not only good efficacy but also good tolerability of atomoxetine was demonstrated in this study and could be confirmed in literature (Spencer et al. 2002; Wernicke and Kratochvil 2002; Biederman et al. 2002; Buitelaar et al. 2004). In contrast to the weight-normalized dose, serum concentrations were in a comparable range regardless of the severity of side effects. Despite a significantly lower weight-normalized dose, patients with severe side effects had serum concentrations comparable to those of patients with minor and moderate side effects. However, time intervals were again initially not considered and could, therefore, lead to bias.

After backcalculating concentrations to the time point of 90 min, still no correlation seemed to exist between serum concentrations and side effects. However, two extreme high serum concentrations above the therapeutic reference range (Hiemke et al. 2018), one of them with minor and the other one with major side effects, led to misleading high mean values. As the two concentrations correlated with both minor and major side effects, high concentrations were not necessarily associated with severe side effects. Furthermore, the other two concentrations with severe side effects and a low weight-normalized dose were even below the therapeutic reference range for adults (Hiemke et al. 2018). Michelson et al. (2007) and Trzepacz et al. (2008) also described a tolerability that was independent of the level of serum concentrations.

The outlier with severe side effects, that was backcalculated to a concentration of 243,694.5 ng/ml at the time point of 90 min, was originally measured with 318 ng/ml at the time point of 36 h after atomoxetine intake. The weight-normalized dose was 0.5 mg/kg. According to the half-life of atomoxetine, serum concentrations should no longer be detectable after 36 h (Witcher et al. 2003; Sauer et al. 2005). Therefore, as assumed earlier, this patient may be defined as a PM. PM can have potentially increased atomoxetine concentrations and extended half-lives compared to EM (Farid et al. 1985; Sauer et al. 2003; Brown et al. 2016). However, genotyping was not performed in this naturalistic study to confirm this assumption. With the assumption of a PM, the formula for backcalculation to the time point of 90 min cannot be applied retrospectively for this patient. The half-life of EM was used in the formula.

Altogether, side effects often seem to occur independently of the level of serum concentrations. However, as described for the concentration of 318 ng/ml after 36 h, they can be associated with elevated serum concentrations in individual cases. Therefore, it is important to detect side effects in time and, despite a generally good tolerability, to always carry out a TDM in children and adolescents as it is requested in the consensus guidelines (Hiemke et al. 2018).

Serum concentrations with minor or no side effects, which represented 80% of all serum concentrations, were both above, within and below the therapeutic reference range valid for adult patients (Hiemke et al. 2018). Due to a nonlinear correlation between serum concentrations and side effects, the suggested therapeutic reference range from 100 to 400 ng/ml for children and adolescents could not be further optimized. Serum concentrations above the upper limit of 400 ng/ml did not necessarily appear to be associated with severe side effects and were well tolerated. Thus, higher serum concentrations could be individually accepted. Not only for atomoxetine but also for other psychotropic drugs with a wide therapeutic window, it is difficult to determine an upper limit (Gründer et al. 2014).

## Study limitations

Naturalistic studies such as this one have some typical limitations (Taurines et al. 2013). Uncontrolled conditions of the clinical setting and potential confounding factors describe two of the limitations. Data were only evaluated retrospectively. Non-compliance and a non-standardized time interval between the intake of atomoxetine and blood collection were only two confounding factors that could cause intraindividual fluctuations in serum concentrations. Serum concentrations with a comedication or a nicotine abuse to name further confounding factors were excluded to assess more valid data. Precise questionnaires like the CGI Severity Scale CGI-S or the ADHD Rating Scale for a more valid assessment of the efficacy and side effects were not available.

Furthermore, due to an incomplete clinical documentation, serum concentrations with missing data for pharmacokinetic analysis had to be excluded and, therefore, sample sizes were very small.

In addition, a quantification of the main metabolites 4-hydroxyatomoxetine and N-desmethylatomoxetine could not be performed due to the lack of the pure substance and an internal standard. This would have been helpful to determine the genotype in case of deviating serum concentrations.

## Conclusion

A significant correlation between weight-normalized dose and serum concentrations of atomoxetine, however, with a small sample size, was first described in this study. The therapeutic reference range proved to be narrower than the therapeutic reference range for adult patients. However, due to good efficacy and tolerability which were both not related to serum concentrations, an exact therapeutic reference range remained difficult to establish for children and adolescents. Individually, higher serum concentrations with a good tolerability seemed to be acceptable. The time window of maximum concentrations during which blood samples should be collected seemed to be similar to the previously defined interval for adult patients. Due to a greater flexibility and better compliance, an extension to a time window of 1–3 h could be discussed. In this time window, no significant decrease of serum concentrations could be demonstrated. To verify and strengthen these preliminary results of this study, further data need to be collected in controlled studies with a larger sample size.

## Data Availability

The data that support the findings of this study are available from the corresponding author, CF, upon reasonable request.
